# A phase I and II study of 2-weekly irinotecan with capecitabine in advanced gastroesophageal adenocarcinoma

**DOI:** 10.1038/sj.bjc.6603084

**Published:** 2006-04-11

**Authors:** M E Burge, D Smith, C Topham, D P Jackson, D A Anthoney, F Halstead, M T Seymour

**Affiliations:** 1 Cancer Research UK Clinical Centre, University of Leeds, Cookridge Hospital, Leeds, UK; 2 Clatterbridge Hospital, Liverpool, UK; 3 St Luke's Cancer Centre, Royal Surrey County Hospital, Guildford, Surrey, UK

**Keywords:** irinotecan, capecitabine, gastric cancer, esophageal cancer

## Abstract

We investigated 2-weekly intravenous irinotecan combined with oral capecitabine in patients with advanced gastroesophageal adenocarcinoma. In phase I, doses were escalated in chemotherapy naïve or pretreated patients to establish maximum tolerated doses (MTD). In phase II, patients were treated at MTD as first-line therapy with the primary end point of RECIST response. Dose levels in phase I were as follows: Level 1: irinotecan 150 mg m^−2^ on day 1; capecitabine 850 mg m^−2^ 12-hourly on days 1–9. Level 2: as level 1 but capecitabine 1000 mg m^−2^. Level 3: as level 2 but irinotecan 180 mg m^−2^. Level 4: as level 3 but capecitabine 1250 mg m^−2^. In phase I, 21 patients were entered. Maximum tolerated dose was level 3. Dose-limiting toxicities were lethargy, diarrhoea, vomiting and mucositis. In phase II, 31 patients were entered at level 3. During the first six cycles, 13 of these patients underwent dose reduction and three patients stopped treatment for toxicity. A further six patients stopped for progressive disease. The commonest grade 3–4 toxicities were lethargy (20%), diarrhoea (17%), nausea (10%) and anorexia (10%). There were no treatment-related deaths. The response rate was 32% (95% CI 16–52%). Median overall survival was 10 months. This regimen is active in gastroesophageal adenocarcinoma. However, using the MTD defined in phase I, fewer than 50% patients tolerated six cycles without modification in phase II; therefore, modification of these doses is recommended for further study.

Gastroesophageal cancer is one of the commonest malignancies worldwide, with around 1.5 million new cases diagnosed in 2002. In the same year, it accounted for over a million deaths, second only to lung cancer (Globocan, 2002). The incidence of adenocarcinoma of the gastroesophageal junction as well as the surrounding distal oesophagus and proximal stomach is rising rapidly in the western world, particularly in younger (40–70 years) white males (Brown and Devesa, 2002). Palliative chemotherapy for locally advanced or metastatic disease prolongs survival and improves quality of life over best supportive care (Murad *et al*, 1993). However, responses tend to be of short duration and median survival is generally less than 1 year. There is no international gold standard regimen, but ECF (epirubicin; cisplatin; protracted infusional 5-fluorouracil (FU)) is accepted as a reference regimen in the UK and Europe, having proven superior to a previous standard regimen, FAMTX (FU, adriamycin and methotrexate) in a phase III randomised trial (Webb *et al*, 1997). More recently, this regimen has been demonstrated to improve long-term survival when added to surgery for operable disease (Cunningham *et al*, 2005). Thus, chemotherapy is becoming increasingly important in the management of this disease at all stages.

However, the ECF regimen is burdensome to the patient, usually entailing an overnight hospital stay, a central line and ambulatory pump for drug delivery, all impacting upon patients' quality of life. Cisplatin is nephro- and neurotoxic and may cause significant asthenia, whereas epirubicin, which makes an uncertain contribution to the regimen, is associated with alopecia, mucositis and cardiotoxicity. The investigation of alternative chemotherapeutic regimens is therefore desirable.

Irinotecan causes DNA damage through interaction with toposoimerase I, and is active in a number of malignancies. In colorectal cancer, it forms part of standard first-line therapy for metastatic disease, where its optimum use, both in terms of activity and toxicity, appears to be fortnightly in combination with infusional FU and leucovorin (LV) (Douillard *et al*, 2000; Tournigand *et al*, 2004; Seymour, 2005). In advanced gastroesophageal cancer, irinotecan has been studied in phase II trials as a single agent and in combination with various other agents, including FU and cisplatin. Single-agent response rates are around 20%, although rates of up to 60% have been reported for irinotecan combined with cisplatin (Kohne *et al*, 2003; Wohrer *et al*, 2004).

Capecitabine is an orally bioavailable FU prodrug, which offers effective fluoropyrimidine therapy while avoiding prolonged intravenous infusion. The final step in its conversion to FU is catalysed by thymidine phosphorylase, an enzyme that is overexpressed in gastric cancers (Miwa *et al*, 1998), which could potentially offer a therapeutic index advantage for capecitabine over FU. The drug is widely used in colorectal cancer where it has proven at least equivalent efficacy but a favourable toxicity profile when compared to bolus intravenous FU/LV in randomised phase III studies (Van Cutsem *et al*, 2004; Twelves *et al*, 2005). It also appears active in gastroesophageal adenocarcinoma. Phase II studies of capecitabine given as a single agent or in combination with a variety of other active agents have been carried out and produced promising response rates (Hong *et al*, 2004; Lorenzen *et al*, 2005). Preliminary data from the randomised ‘REAL 2’ trial in this disease show that capecitabine can be substituted for infusional FU as part of the ECF regimen without loss of efficacy (Sumpter *et al*, 2005). Preclinical data suggest that capecitabine and irinotecan may act synergistically (Hapke *et al*, 2001).

Thus, there is good reason to investigate the combination of irinotecan and capecitabine in the management of gastroesophageal adenocarcinoma. However, the optimum schedule for this combination has not yet been established. Studies in colorectal cancer have most commonly been based on the standard single-agent capecitabine schedule of 14 days treatment every 3 weeks, with irinotecan added as a single 3-weekly administration. However, the tolerability of this regimen is uncertain (Kohne *et al*, 2005). We therefore decided that, while evaluating these drugs in gastroesophageal cancer patients, we would take the opportunity to explore a different schedule, modelled on the attractive 2-weekly FOLFIRI regimen now well established in colorectal cancer. Capecitabine was administered on days 1–9 of the 14-day cycle in order to maintain dose density comparable with standard capecitabine schedules. The aims of this phase I/II study were to define the optimum doses of the combination and to obtain preliminary efficacy data.

## PATIENTS AND METHODS

The study included phase I and phase II components. Phase I was open to chemo-naïve or pretreated patients, and incorporated both between-cohort and within-cohort dose escalation in order to establish optimum doses of both drugs. In phase II, these doses were evaluated for efficacy and tolerability, over a 12-week treatment period, in a further 31 patients receiving the regimen as first-line treatment for advanced disease.

Recruitment to the phase I component of the study was from a single institution and started in September 2001. Two further centres were involved in recruitment to the phase II component, which completed in November 2002. Ethical approval was obtained at all participating institutions. To be eligible for either phase, patients had to have histologically confirmed locally advanced or metastatic adenocarcinoma of the oesophagus or stomach, unsuitable for curative surgery or chemoradiotherapy; age >18 years; WHO performance status 0, 1 or 2; adequate haematological and biochemical parameters (white blood cells ⩾4, platelets ⩾150, bilirubin ⩽1.5 × upper limit of normal (ULN), alkaline phosphatase <5 × ULN, aspartate aminotransferase and alanine aminotransferase <2.5 × ULN and creatinine clearance >50 ml min^−1^); and written, informed consent. Main exclusion criteria were as follows: prior radical chemoradiotherapy; treatment with radiotherapy or any investigational drug within the preceding 4 weeks; a prior severe reaction to fluoropyrimidine therapy; concurrent uncontrolled medical condition (e.g. angina and cardiac failure), infection or diarrhoea; requirement for a contraindicated medication; history of epilepsy; any condition that may impair oral self-medication; pregnant or lactating women, or inadequate contraception in women of childbearing potential.

In addition to these general selection criteria, patients in the phase II study were required to have measurable disease, and may not have received prior palliative chemotherapy for locally advanced/metastatic disease. Prior (neo)adjuvant, chemotherapy was allowed if completed at least 3 months before randomisation.

### Pretreatment evaluation and study assessments

Clinical evaluation and investigations to confirm eligibility were to be completed within 1 week before starting therapy. Investigations to measure disease, such as endoscopy, computed tomography (CT) and/or magnetic resonance imaging, were required within 3 weeks. On the first day of each treatment cycle, patients were evaluated for toxicity using the NCIC-CTC version 2 and blood was drawn for full blood count (FBC), renal and liver function. Response to treatment was formally assessed following cycle 6 (weeks 13–14). Standard RECIST criteria were used to define a partial or complete response, stable disease or disease progression. Responses required confirmation with repeat assessment at least 4 weeks later.

### Treatment of patients

Irinotecan was administered at doses ranging from 150 to 180 mg m^−2^ in 250 ml normal saline over 1 h. Capecitabine was administered at doses ranging from 850 to 1250 mg m^−2^ (to the nearest dose achievable using 500 and 150 mg tablets). The first dose was taken at the end of the irinotecan infusion, the second at 2100 the same evening, and thereafter at 0900 and 2100, within 30 min of ingestion of food, on days 1–9 (total 18 doses). Cycles were repeated every 14 days. Conventional supportive measures for nausea/vomiting, anticholinergic syndrome and diarrhoea were employed. Protocol-specified standard dose reductions and/or delays were implemented based on toxicity experienced during the previous cycle and pretreatment haematological, renal and hepatobiliary function.

Table 1 shows the four dose levels tested in the dose-escalation phase. At each dose level, patients were recruited until at least four had completed at least three cycles of treatment, but without pauses (so total cohort size could exceed four patients). Recruitment then progressed to the next dose level if 25% or less of four or more patients had experienced a dose-limiting toxicity (DLT) during the first three cycles. In addition, patients who were entered at dose levels 1 or 2 who experienced no toxicity above grade 2 after the first three cycles could be escalated to the next dose level and contribute to the evaluation of that dose. Dose-limiting toxicity was defined as any non-haematological toxicity (excluding alopecia) of grade ⩾3, or haematological toxicity of grade 4 causing a chemotherapy delay of more than 5 days. Maximum tolerated dose (MTD) was defined as one dose level below that causing DLT in >25% of four or more patients. This was the dose taken forward to the phase II component of the study.

Study treatment was planned to last 12 weeks (six cycles) at which time the primary efficacy end point of response rate was assessed. Patients who had stable or responding disease at this time could, at the investigators discretion, then continue on the same regimen, either immediately or after a break. Any treatment beyond six cycles, however, did not contribute to efficacy assessment. Patients could also be considered, at this time, for surgery or radiotherapy if appropriate. Study treatment was discontinued in the event of disease progression during chemotherapy, intolerable adverse events or at the patient's request. Second-line therapy with ECF was available when appropriate.

### Statistical considerations

It was planned to recruit 30 patients to the phase II study. This sample size provides a reasonable estimate of the response rate upon which to base further study design. For example, 15 (50%) responders would give a true response rate estimate of 31–69% (*α*_1_=2.5%; *α*_2_=5%; *γ*=95% binomial sampling distribution; Neave, 1981). An early stopping rule would have closed the study after 14 patients if no RECIST responses had been seen as this would indicate a 95% probability of the true response rate being <20%.

Overall survival was calculated from the date of first chemotherapy administration to the date of last follow-up or death from any cause. Median overall survival was estimated using the Kaplan–Meier method.

## RESULTS

Twenty-one patients were recruited to the phase I dose-escalation study and 31 to the phase II study. Table 2 summarises their pretreatment characteristics. The majority were male, had good performance status and had the primary tumour *in situ*. Half were symptomatic. The median age for the combined cohorts was 61 years. The vast majority were chemotherapy naïve. Three patients in phase I had received prior palliative chemotherapy and one had received prior preoperative chemoradiotherapy. One patient in phase II had previously received neoadjuvant chemotherapy.

### Phase I

Table 3 summarises the DLTs experienced.

Five patients were recruited to dose level 1. Dose-limiting toxicity occurred in one patient (grade 3 lethargy) after the first cycle, who declined further treatment. Two other patients tolerated three cycles but came off study at that point, one because of progressive disease and the other died as a result of chronic obstructive airways disease, unrelated to treatment. The other two patients were escalated to dose level 2, one of whom died from a cerebrovasular accident, not attributed to chemotherapy, after one further cycle.

Six patients were entered at dose level 2. One developed DLT (grade 3 mucositis and diarrhoea) and discontinued treatment after two cycles. The other five patients all completed at least three cycles and three were escalated to dose level 3. Two patients required a non-DLT-related dose reduction or delay, hence were not escalated. No DLTs occurred in the six patients either entered at, or escalated to, dose level 3, all of whom completed at least three cycles at the intended dose. At dose level 4, six out of seven patients experienced a DLT (Table 3) and all seven required a dose reduction.

Dose level 3 was therefore declared the MTD and patients recruited to the phase II study were treated at this dose. No haematological DLTs were encountered at any dose level.

### Phase II

#### Toxicity

Thirty-one patients were entered into the phase II study, but one patient had disease progression and died before receiving any study therapy. A total of 165 cycles were administered to the remaining 30 patients of which 117 (71%) were at the full dose. Patients received a median of six cycles (range 0–12). Chemotherapy was delayed in 22 (13%) of cycles. The majority of patients, 19 (63%), completed at least six cycles but of these eight (42%) required a dose reduction. Hence, 11 patients (33%) completed six cycles at the full dose whereas the vast majority (86%) received two cycles and 74% received three cycles at full dose. During the first six cycles, 13 patients required a dose reduction, a further three stopped therapy owing to toxicity, six withdrew owing to progressive disease and a further two patients died. One of these two patients had a fatal pulmonary embolus and the other died of disease progression. There were no treatment-related deaths.

Table 4 summarises the toxicity experience per patient and Table 5 shows the distribution of the worst toxicity grades experienced.

Forty-two per cent of the phase II cohort experienced grade ⩾3 toxicity at some stage during their treatment. The commonest grade 3 toxicities were lethargy (19%), diarrhoea (16%), nausea (10%) and anorexia (10%). Haematological toxicity was low. One patient developed grade 4 neutropenia in association with grade 3 diarrhoea, but no neutropenic fever/infection was encountered.

#### Efficacy

Table 6 summarises the efficacy results. Of the 31 patients recruited, three were not assessable for response: one patient progressed before receiving any treatment and two withdrew after only one cycle of chemotherapy owing to toxicity. Nine (32%) of the 28 assessable patients had RECIST partial responses and an additional nine patients (32%) achieved stable disease for at least 12 weeks. If the non-assessable patients are included for an ‘intent to treat’ analysis, these rates fall to 29% partial responses and 29% stable disease. The median overall survival for the phase II cohort was 10 months. Six of the 16 (38%) assessable patients recruited to the phase I study responded, including one complete response.

## DISCUSSION

The combination of irinotecan and capecitabine is a reasonable regimen to explore in advanced gastroesophageal cancer: both drugs are known to be active; there is preclinical evidence of synergy between them (Hapke *et al*, 2001); their toxicities only partially overlap, and the regimen avoids central lines and infusion pumps. In addition, there does not appear to be any significant pharmacokinetic interaction between the two drugs (Tewes *et al*, 2003). There are several published phase I and II studies exploring this combination in advanced colorectal cancer but little published data in gastroesophageal cancer. The investigation of a 2-weekly regimen is warranted. In metastatic colorectal cancer, 2-weekly irinotecan and infusional FU has been extensively investigated and is a well-established regimen based on its favourable efficacy and toxicity profile (Douillard *et al*, 2000; Tournigand *et al*, 2004). In a large randomised trial, again in colorectal cancer, when used as second-line therapy, 2-weekly irinotecan/infusional FU was associated with significantly less diarrhoea and alopecia compared with 3-weekly single-agent irinotecan (Seymour, 2005). Moreover, a 3-weekly irinotecan/capecitabine schedule was associated with significant toxicity in a recent randomised study from the EORTC (Kohne *et al*, 2005). Our 2-weekly schedule was designed to achieve similar dose intensities to the 3-weekly schedule investigated in colorectal cancer. (Borner *et al*, 2005; Kohne *et al*, 2005).

The phase I component of our study established dose level 3 (irinotecan 180 mg m^−2^ with capecitabine 1000 mg m^−2^ b.d. on days 1–9) as the MTD. None of the six patients treated at this dose experienced a DLT, whereas six out of seven patients treated at dose level 4 experienced DLT, principally diarrhoea, lethargy and vomiting. We observed little haematological toxicity and no cases of febrile neutropenia, but routine mid-cycle FBC was not performed. Hand–foot syndrome (HFS) above grade 2 was not encountered in our phase I cohort. By comparison, in phase I studies of this combination in colorectal cancer, using a 3-week cycle, the principal toxicities were diarrhoea, lethargy and vomiting, but also neutropenia (Tewes *et al*, 2003; Delord *et al*, 2005; Rea *et al*, 2005).

Dose level 3 was used in the first-line setting to treat an additional cohort of patients in the phase II study. Although 74% of these patients were able to receive at least three cycles at the full dose, toxicity was nonetheless significant and greater than experienced during phase I, with 16 out of 30 (53%) patients eventually having a dose reduction or withdrawal during the first six cycles. The most common toxicities were, once again, lethargy, diarrhoea, nausea and anorexia occurring at any grade in 77, 67, 59 and 45% of patients, respectively and reaching grade ⩾3 in 19, 16, 10 and 10%, respectively. Although in this patient population such symptoms may have been only partly caused by the chemotherapy drugs, in many patients, they were cyclical in nature and it was felt that therapy was a significant contributor. There were no episodes of febrile neutropenia, and grade >2 HFS occurred in only two (6%) patients. We noted three acute venous events during treatment, although none were reported by the clinicians as treatment related. A possible explanation for the greater toxicity seen for dose level 3 in phase II is that the intra-patient dose escalation utilised during phase I introduced selection bias (patients tolerating lower dose levels are more likely to tolerate higher dose levels than treatment-naïve patients) and consequently underestimated the toxicity of dose level 3.

Published phase II studies of the irinotecan/capecitabine combination in colorectal cancer have reported similar frequencies and types of grade ⩾3 toxicity, with diarrhoea being the commonest, occurring in around 20% of patients and grade ⩾3 neutropenia, lethargy, nausea and anorexia in around 10–20% (Bajetta *et al*, 2004; Borner *et al*, 2005). Recently, the EORTC 40015 phase III study, comparing 2-weekly irinotecan/LV5FU2 *vs* 3-weekly irinotecan/capecitabine (both arms±celecoxib) in advanced colorectal cancer, was stopped prematurely because of excessive toxicity (Kohne *et al*, 2005). In that study, there was significantly more grade ⩾3 diarrhoea with irinotecan/capecitabine (31%) than with irinotecan/LV5FU2 (6.9%); in addition, deaths felt to be caused or exacerbated by treatment occurred in five of 44 (11%) patients on irinotecan/capecitabine, most commonly with diarrhoea, sepsis and/or thrombotic events. The investigators concluded that the doses used (irinotecan 250 mg m^−2^; capecitabine 1000 mg m^−2^ b.d. on days 1–14 every 3 weeks) were not feasible in their study.

Our 2-weekly regimen is active in advanced gastroesophageal adenocarcinoma in the first-line setting. In the phase II cohort, the objective response rate was 32%, and an additional 14% patients with stable disease by RECIST criteria nonetheless experienced symptomatic improvement together with improvement in CT scan appearances and/or serum markers. Although similar to the reported results of standard ECF or other irinotecan-based regimens, comparison is of course not possible in this non-randomised study.

We used a single (weeks 13–14) time point at which to assess response. This may have underestimated the response rate compared with a method using serial imaging to assess the best response obtained. However, in our experience, the vast majority of patients with responsive gastroesophageal adenocarcinoma will have responded following 3 months of effective therapy.

In colorectal cancer, it has been shown that, when combined with 2-weekly oxaliplatin, administered dose intensity of capecitabine can be increased without additional toxicity when compared to the standard 3-weekly schedule. This was achieved by administering the fluoropyrimidine at higher dose for 7 days during each 14-day cycle. Additionally, this regimen was associated with increased response rates and time to progression (Scheithauer *et al*, 2003). It is conceivable that, by adopting a similar schedule in combination with 2-weekly irinotecan, the efficacy of our regimen could be improved. However, it is quite possible that the toxicity of this dose-intensive regimen in combination with irinotecan, in patients with gastroesophageal cancer, would be prohibitive.

Recently, a phase II trial of a 3-weekly regimen of irinotecan/capecitabine in advanced gastroesophageal carcinoma was presented in abstract form (Assersohn *et al*, 2005). This regimen was administered to 25 eligible patients who had all progressed through, or relapsed within 3 months of, platinum-based chemotherapy. Of the 21 assessable patients, three (14%) responded and a further three (14%) had stable disease. An improvement in a variety of tumour-related symptoms was also noted. Only one patient (4%) developed febrile neutropenia and there were no toxic deaths, but further toxicity data are yet to be reported.

Irinotecan combined with infusional FU/LV has been studied in two recent randomised phase II studies. In the first, this combination, given using a weekly schedule, appeared to provide superior efficacy to irinotecan/cisplatin, but with a high rate (27%) of grade ⩾3 diarrhoea (Pozzo *et al*, 2004). In the second, the fortnightly irinotecan/FU/LV regimen was compared to FU/LV alone and FU/LV/cisplatin: it produced better rates of response and progression-free survival than either comparator (Bouché *et al*, 2004). The response rate and median overall survival seen with irinotecan/FU/LV in these two studies was 42%, 10.7 months and 40%, 11.3 months, respectively, consistent with our findings for irinotecan/capecitabine. Most recently, a phase III trial comparing irinotecan/FU with cisplatin/FU has been presented in abstract form: there was a non-statistically significant trend towards improved time to progression in the irinotecan arm but there was no difference in overall survival; patients randomised to irinotecan/FU experienced less febrile neutropenia, nausea and stomatitis but more diarrhoea than those randomised to cisplatin/FU (Dank *et al*, 2005).

Based on the data presented to date, it appears that irinotecan-based regimens represent a treatment option for the treatment of gastroesophageal cancer, but are not a major therapeutic advance over standard cisplatin-based regimens. They seem to have similar efficacy, and represent a useful option, especially for patients at risk of cisplatin toxicities. They may also represent a treatment option in the second-line setting following failure of cisplatin, but ideally this should be tested in a randomised trial against best supportive care. It is uncertain at this stage if capecitabine can be substituted for FU without compromising efficacy and safety when used in combination with irinotecan; our experience would suggest that this is feasible, but the safety results of EORTC 40015 in advanced colorectal cancer are a cause for concern.

In conclusion, our study suggests that the fortnightly regimen of irinotecan 180 mg m^−2^ on day 1 together with capecitabine 1000 mg m^−2^ on days 1–9 is an active regimen in advanced gastroesophageal adenocarcinoma, but requires modification in order to improve its toxicity profile. In the MRC FOCUS trial in advanced colorectal cancer, this regimen – but with a reduced capecitabine dose of 800 mg m^−2^ b.d. on days 1–9 – was used as second/third-line crossover treatment after FU/LV and oxaliplatin (Seymour, 2005). To date, it appears to be active and well tolerated in that context, but full reporting of that trial is awaited.

## Figures and Tables

**Table 1:** Dose-escalation schedule

**Level**	**Irinotecan dose (1 h i.v. infusion, day 1) (mg m^−2^)**	**Capecitabine dose (p.o., every 12 h, days 1–9) (mg m^−2^)**
1	150	850
2	150	1000
3	180	1000
4	180	1250

**Table 2:** Patient characteristics (number (%))

	**Phase I**	**Phase II**	**Total**
	***N*=21**	***N*=31**	***N*=52**
Median age (range)	57 (40–71)	63 (40–78)	61 (40–78)

*Gender*			
Male	18	24	42
Female	3	7	10

*WHO performance status*			
0	7 (33)	10 (32)	17 (33)
1	14 (66)	16 (52)	30 (57)
2	0	5 (16)	5 (10)
Symptomatic	12 (57)	15 (48)	27 (51)

*Primary tumour site*			
Stomach	8 (38)	18 (58)	26 (50)
OG junction	4 (19)	7 (23)	11 (21)
Oesophagus	9 (43)	6 (19)	15 (29)
Primary tumour resected	3 (14)	6 (19)	9 (17)
Measurable disease	16 (76)	31 (100)	—

*Sites of metastases*			
Local/anastomotic	0	9 (29)	9 (17)
Liver	9 (43)	14 (45)	23 (44)
Peritoneum	5 (24)	15 (48)	19 (37)
Lung	3 (14)	1 (3)	4 (8)
Lymph nodes	8 (38)	14 (35)	19 (37)
Other	3 (14)	8 (26)	11 (21)

OG=oesophageal-gastric junction.

**Table 3:** DLTs experienced at four dose levels during the dose-escalation phase

**Dose level**	**1**	**2**	**3**	**4**
Irinotecan (mg m^−2^)/capecitabine(mg m^−2^ b.d.)	150/850	150/1000	180/1000	180/1250
Patients entered at dose level (*n*)	5	6	3	7
Patients escalated from previous dose level (*n*)	—	2	3	—
Total patients treated (*n*)	5	8	6	7
Number completing at least three cycles at intended dose	4	6	6	5

*DLTs* (*n* (%))				
Lethargy grade 3	1 (20)			3 (43)
Diarrhoea grade 3		1 (17)		1 (14)
Diarrhoea grade 4				1 (14)
Mucositis grade 3		1 (17)		
Vomiting grade 3				1 (14)

b.d.=twice daily; DLT=dose-limiting toxicity.

**Table 4:** Maximum toxicity grade per patient (number (%); *n*=30)

**Toxicity**	**Grade 1**	**Grade 2**	**Grade 3**	**Grade 4**
Anaemia	—	1 (3)	—	—
Neutropenia	—	1 (3)	2 (7)	1 (3)
Lethargy	7 (23)	11 (37)	6 (20)	—
Diarrhoea	10 (33)	6 (20)	5 (17)	—
Nausea	7 (23)	8 (27)	3 (10)	—
Vomiting	5 (17)	7 (23)	2 (7)	—
Mucositis	7 (23)	3 (10)	—	—
Alopecia	7 (23)	8 (27)	—	—
Anorexia	5 (17)	6 (20)	3 (10)	—
Skin toxicity	4 (13)	1 (3)	2 (7)	—
Ataxia	—	1 (3)	1 (3)	—
Neuropathy	2 (7)	—	—	—
Abdominal pain	5 (17)	1 (3)	—	—
Dyspepsia	4 (13)	—	—	—
Constipation	3 (10)	—	—	—
Fever	1 (3)	1 (3)	—	—
Infection	1 (3)	—	—	—
Cholinergic	3 (10)	—	—	—
Raised bilirubin	—	—	1 (3)	—
Hiccoughs	—	—	1 (3)	—
Other	6 (20)	3 (10)	—	—

**Table 5:** Worst toxicity experienced (*n*=30)

**Worst toxicity grade**	**Number of patients (%)**
0	4 (13)
1	1 (3)
2	13 (43)
3	12 (40)
4	1 (3)

**Table 6:** Efficacy results (response rate) by phase I and II cohorts and for all assessable patients combined (*n* (%) (95% confidence interval))

	**Phase I**	**Phase II**	**All patients**
Complete/partial response	6 (38) (15–65)	9 (32) (16–52)	15 (34) (20–50)
Stable disease	5 (31) (11–58)	9 (32) (16–52)	14 (32) (19–48)
Progressive disease	5 (31) (11–58)	10 (36) (19–56)	15 (34) (20–50)
Number of assessable patients	16	28	44
